# Expert nurses’ coping strategies in ethically challenging situations: a qualitative study

**DOI:** 10.1186/s12912-021-00709-w

**Published:** 2021-09-29

**Authors:** Yeon Hee Kim, Young-ah Kang, Jeong Hui Ok, Kwisoon Choe

**Affiliations:** 1 Department of Clinical Nursing, University of Ulsan, 88, Olympic-ro 43-gil, Songpa-gu, Seoul, South Korea; 2 Hematology Unit, Asan Medical Center, 88, Olympic-ro 43-gil, Songpa-gu, Seoul, South Korea; 3 Cancer Emergency Room, Asan Medical Center, 88, Olympic-ro 43-gil, Songpa-gu, Seoul, South Korea; 4 Department of Nursing, Chung-Ang University, 84 Heukseok-ro, Dongjak-gu, Seoul, Republic of Korea

**Keywords:** Coping behavior, Ethical competency, Healthcare ethics, Medical ethics, Nursing ethics, Professional ethics

## Abstract

**Background:**

Nurses frequently encounter ethically challenging situations in everyday practice. In these situations, nurses often know an appropriate course of action to take but are unable to do so. Many studies have examined the ethically challenging situations faced by nurses, but how nurses cope with these situations is not well understood. Therefore, this study aims to explore the coping strategies used or adopted in ethically challenging situations by expert nurses in South Korea.

**Methods:**

Participants were recruited via purposive sampling. Small group interviews were conducted with 26 expert registered nurses in a general hospital in South Korea. The data were analyzed using Giorgi’s descriptive phenomenological method.

**Results:**

The essential theme of nurses’ experience of coping with ethically challenging situations was “being faithful to the nature of caring.” This essential theme comprised three themes: self-monitoring of ethical insensitivity, maintaining honesty, and actively acting as an advocate.

**Conclusions:**

The findings of this study suggest that the coping strategies of expert nurses are mostly consistent with the attributes of ethical competence as previously defined in healthcare, and expert nurses can address ethically challenging situations in an effective and ethical manner by faithfully adhering to the spirit of caring. System-wide early counseling and interventions should be considered for nurses who have experienced ethical difficulties.

## Background

Ethically challenging situations that nurses encounter in everyday practice are usually situations in which they know an appropriate course of action to take but do not do so [1]. Sometimes, nurses may have difficulty in recognizing the right course of action. Historically, the medical field has lacked a clear definition of an ethically challenging situation in nursing. However, studies within the last decade have explored the ethically challenging situations that registered nurses’ experience in nursing practice. For example, nurses in the emergency department reported that ethically challenging situations involve being close to the suffering or death of people, being unsure how to express their feelings, having a heavy responsibility, and working in an open space with little privacy for their work with patients [2]. In addition, nurses providing childhood cancer care in Sweden were often concerned about infringing on patients’ autonomy, deciding on the appropriate treatment level, and dealing with conflicting perspectives within health professional teams [3].

What nurses require is the ethical competence to cope with such ethically challenging situations. Ethical competence can be defined in terms of individuals’ character strength (good character traits), ethical awareness (ethical perception), moral judgment skills, and willingness to do the right thing [4]. Ethical competence depends on the ability to detect ethically challenging situations, consider various courses of action, and implement them [1]. Consequently, ethical competence results in positive outcomes for the patient and reduces nurses’ moral distress [4], and exploring how nurses cope with ethically challenging situations could improve understanding of nurses’ ethical competence.

Numerous studies worldwide have examined ethically challenging situations among nurses in clinical settings. Rathert et al. [5] found that more than half of the 290 nursing staff surveyed in acute care hospitals in the United States experienced ethical dilemmas and conflicts frequently (i.e., several times a month to daily). Healthcare professionals in Sweden, including nurses, described three themes of ethically difficult situations: a sense of powerlessness in managing the complex emotional needs of patients and relatives, providing unequal care, and uncertainty over who is the primary care decision-maker [6]. A study in South Korea found that conflicts between nurses and physicians or other nurses were the most frequently encountered ethically challenging situations across hospital departments [7]. Ethically challenging situations can lead to moral distress in nurses [5, 8, 9], which can, in turn, directly harm patients as well as nurses’ personal and professional lives [10]. Therefore, it is crucial for nurses to cope effectively with ethically challenging situations.

In reviewing the literature, the authors found a few studies [8, 9, 11, 12] that reported on nurses’ coping strategies for dealing with ethically challenging situations. For example, nurses in a hemodialysis center tried to discuss ethical concerns with physicians but largely failed in doing so, causing them to continually feel uncertain and act against their conscience [11]. Oncology nurses mainly remained silent about ethical concerns, which shaped a culture of avoidance in conversations on the prognosis and end-of-life treatment with patients, families, and physicians [12]. The literature review of coping strategies [9] suggested that nurses use various coping resources, which can be positive when they lead to dialogue and reflection or negative when they cause the professional to accept and conform to the context, experience difficult ethical situations alone without the support of colleagues or the institution, or become prone to a feeling of moral distress. These previous studies contribute to our understanding of how nurses deal with ethically challenging situations. However, three of the previous studies [8, 11, 12] narrowly focused on nurses’ coping strategies in special units such as hemodialysis or oncology and did not explore the strategies used in general units, and two studies [8, 9] reported coping strategies for moral distress but did not focus on the ethically challenging situations themselves. Overall, the previous studies did not fully explore how nurses deal with ethically challenging situations in nursing practice. In the current study, the authors were interested in expert nurses’ ways of coping because the nurses have abundant experience in dealing with ethically challenging situations. Therefore, this study aims to explore the experiences of expert nurses with regards to coping strategies used or adopted in ethically challenging situations in South Korea.

## Methods

### Design

This study used a descriptive phenomenological research method, as suggested by Giorgi [13], which is suited for describing an individual’s perspective on their experience.

### Study setting

The authors selected a general hospital located in the capital city of South Korea for this study because it is known as the first and best hospital with a systematic career evaluation system for nurses. The career evaluation system of this general hospital assesses and develops the careers of nurses according to four stages: 1) a nurse complies with nursing standards and performs nursing tasks without difficulty, 2) a nurse adjusts nursing goals and priorities and plays the role of a preceptor, 3) a nurse is an expert who can individualize and manage the nursing needs of a variety of situations, and 4) a nurse is a head nurse or a professional clinical nurse who is capable of being a role model for nurses [14].

### Participants

To recruit expert nurses in clinical settings through purposive sampling, nursing team managers or unit managers were asked to recommend expert nurses whose expertise was determined using the career evaluation system. In South Korea, nursing team managers or unit managers, including head nurses, spend more time doing administrative work than providing direct nursing care for patients. Therefore, the authors recruited expert nurses who directly care for patients in their practice, and also tried to recruit nurses working in a variety of wards to gain the broadest perspective on the ethically challenging situations nurses face. Recommended expert nurses were invited to this study, and 26 registered nurses participated voluntarily.

### Data collection

All data were collected through six small group interviews, and each group interview took place twice. Each group consisted of four to five participants, and the participants in each group remained unchanged until the end of the data collection. The authors chose the small group interview because it helps to “create a natural communicative context for telling stories from practice, allowing peers to talk to one another as they ordinarily talk” in a phenomenological study ([15], p.109). The participants of this study did not have conflict with or contradict each other but empathized with each other in the group interview. The authors informed each nurse of the main interview question, which was “*How did you cope with ethically challenging situations you experienced while working as a nurse?*” via e-mail or phone 1 week before the group interview. The interview location was a hospital counseling room or a café near the hospital.

At the beginning of the first interview, participants completed a questionnaire asking about their general characteristics, such as their age, sex, job title, educational background, and work experience. The interview began with the question: *“How did you deal with ethically challenging situations while working?”* Then, the authors asked open-ended questions such as, “*Please tell me what you experienced while dealing with ethically challenging situations*.”

One to 2 weeks after the first interview, the second interview was conducted. During the second interview, the authors asked participants additional questions about the first interview that needed further clarification. Each interview was recorded with the consent of the participants and lasted for 2–2.5 h. After interviewing the 26 participants a second time, no new themes were identified indicating data saturation had been achieved, and no additional participants were recruited. The interviews were conducted in Korean, and the data analysis and description of results were in Korean. Then, one of the authors wrote the manuscript in English.

### Ethical considerations

The Institutional Review Board of the hospital bioethics committee approved this study. Participants were informed of the study’s purpose and methods and their right to withdraw participation at any time up until the study was completed. The participants voluntarily signed a written informed consent form. Their privacy and confidentiality were ensured by referring to them in the manuscript by letters rather than their names.

### Data analysis

A phenomenological approach [13] was used to interpret the content of the text data through the systematic coding and identification of themes. The audio-recorded interview data were transcribed verbatim by the authors (K and O). Giorgi [13] recommended a three-tiered analysis. First, all authors read the interview transcripts repeatedly to grasp the overall meaning of the text. In the second step, meaningful statements were selected; two authors (K and O) underlined the sentences or phrases considered most relevant for nurses’ coping with ethically difficult situations. Third, each meaning unit (i.e., the participants’ own words) was transformed into ‘phenomenologically psychologically sensitive expressions’ as described by Giorgi [13]. More specifically, two authors (K and O) summarized the meaning of each sentence using third-person expressions by making a coding list. All authors met several times to review the list of codings and discuss how to group them into more abstract themes. Codings were sorted and categorized into sub-themes based on the similarity of meaning, which were then grouped into themes. For example, awareness of human dignity, self-reflection, and ethical questioning while working were conceptualized into a theme of “self-monitoring of ethical insensitivity.” We then grouped the sub-themes into main themes—self-monitoring of ethical insensitivity, maintaining honesty, and actively correcting mistakes—into the essential theme “being faithful to the nature of caring.”

### Rigor

The study’s rigor was examined in terms of credibility, dependability, and transferability, as described by Graneheim and Lundman [16]. For credibility, we, the authors (one of whom was an expert in qualitative research), discussed the text several times until we all agreed with the way the data were labeled and sorted. Additionally, the authors showed the results of the analysis to several participants who ensured the results of the study matched what the participants experienced. Regarding dependability, authors held discussions throughout the data analytic process to ensure the selection of consistent themes. Finally, to ensure transferability, the authors described the general characteristics of participants and ethical situations in detail for the reader to understand the analytic process.

## Results

All 26 participants were women, and their average age was 36.7 years (range: 32–44 years). They had been employed in the current hospital for an average of 12 years and 1 month (range: 9 years and 9 months to 17 years and ten months; Table 1). The essential theme of expert nurses’ experiences of coping strategies with ethically challenging situations was “being faithful to the nature of caring.” This essential theme, in turn, comprised three themes: self-monitoring of ethical insensitivity, maintaining honesty, and actively acting as an advocate (Table 2).

**Table 1:** General characteristics of participants (*n* = 26)

Characteristics		*n* (%)
Sex	Female	26 (100)
Age (years)		36.7 (32–44)
Education level	Associate degree	3 (11.5)
	Bachelor of Science in Nursing	18 (69.2)
	Master of Science in Nursing	5 (19.3)
Work experience (years)		12.1 (7.10–17.10)
Department	Medical department	8 (30.8)
	Surgical department	8 (30.8)
	Intensive care unit	5 (19.2)
	Operating department	2 (7.7)
	Emergency department	2 (7.7)
	Pediatric department	1 (3.8)
Position	Registered Nurse	14 (53.8)
	Charge Nurse	12 (46.2)

**Table 2:** Themes of ‘being faithful to the nature of caring’

Themes	Sub-themes
1. Self-monitoring of ethical insensitivity	(1) Awareness of human dignity (2) Self-reflecting (3) Ethical questioning
2. Maintaining honesty	(1) Adhering to the principles and standards of nursing practice (2) Internalizing honesty
3. Actively acting as an advocate	(1) Expressing oneself regarding the treatment or erroneous situation (2) Being a mediator

### Theme 1: self-monitoring of ethical insensitivity

The expert nurses in this study continually and consciously monitored their ethical insensitivity when confronted with ethically challenging situations. Self-monitoring of ethical insensitivity was a cyclical process involving three sub-themes: awareness of human dignity, self-reflecting, and ethical questioning.

#### Sub-theme 1: awareness of human dignity

Participants were constantly aware of the human dignity of their patients in the face of ethically challenging situations. This awareness gave participants new insights into their role as a nurse, bringing them a sense of duty in caring for humans.

*“A patient (waiting for organ transplants) was whispering, ‘If I live, someone is dead, right?’... I was busy, and I had not even thought about it. Yes. ... the work of saving someone (through organ transplants) requires someone to have died. It is not a situation that I can ignore using my busyness as an excuse” (Participant A, 11 years of experience, medical department).*

When expert nurses reminded themselves that they were caring for a human, they became worried they might undermine human dignity. In busy and often complicated clinical situations, nurses frequently used patients’ medical diagnosis and room number (e.g., ‘pneumonia, 1203′) instead of patients’ names when talking to other nurses and medical staff, which some believed would help to limit their mistakes in nursing practice; however, they also acknowledged that this practice sometimes led nurses to not treating patients with dignity.

#### Sub-theme 2: self-reflecting

Participants often remembered and self-reflected on moments in which they encountered ethically challenging situations such as when the workload limited their opportunities to express compassion for patients. Moreover, some participants reflected on how nurses lose opportunities to hold a patient’s hand or speak warmly to patients and their relatives, feeling that they had not done their job as nurses in those instances. Some participants reflected on situations where nurses could not talk about the patient’s treatment or prognosis (often on the doctor’s orders). Then, they experienced agony in having to observe helplessly from the periphery, mainly when life-saving treatment was hopeless. *“It’s clear that this patient will not be able to live beyond a few days, but the doctor continues the treatment until the end, and family members want to let the patient go comfortably. I think it is not in the best interest of the patient or his family if he is made to keep breathing, take high doses of drugs, undergo dialysis, and use a ventilator” (Participant Q, 14 years of experience, surgical department).*

#### Sub-theme 3: ethical questioning

Participants asked themselves who their nursing was for and whether they were taking the right or appropriate actions in their nursing practice. Ethical questioning arose in situations in which participants had to keep a secret about a cancer diagnosis, apply physical restraints to patients, and provide care for patients with do not resucitate orders (DNRs). For example, some participants questioned whether it was right not to tell patients with cancer the truth of their diagnosis as requested by the family caregivers or to change the position every two hours to prevent bedsores of patients who are about to die. *“It’s an aging society, and there are a lot of older people whose families do not want them to know about it (their cancer diagnosis). Most of us ensure confidentiality, but is it right to keep it a secret because the patient is so old? Family members may give up treatment because patients are old. Caregivers don’t let patients decide for themselves about treatment—is this right?”(Participant M, 17 years and 10 months of experience, surgical department).*

### Theme 2: maintaining honesty

All participants in this study thought that adhering to the standards of nursing was fundamental to honesty. They strove to be honest by adhering to principles and standards of nursing and internalizing honesty.

#### Sub-theme 1: adhering to principles and standards of nursing practice

Participants realized that adhering to nursing standards and principles was the basis of ethical nursing practice. Many participants considered it unethical for a nurse to not know the principles and standards of nursing practice. They reported that even simple nursing tasks had to be performed faithfully and transparently in accordance with these standards. Examples of these standards were giving medications only as prescribed, not fabricating nursing records, and not acting hypocritically to cover up a problem.

*“In the past, a patient who was waiting for an organ transplant sometimes asked me to record his condition as worse than it was. He asked me to record that he’s unconscious … I told the patient that it wasn’t possible” (Participant P, 11 years and three months of experience, surgical department).*

Moreover, participants rejected doctors’ unscrupulous orders and lacked tolerance for other colleagues’ mistakes. Nurses sometimes encountered conflicts in their relationships with other nurses, doctors, patients, and relatives of patients while adhering to these principles.

#### Sub-theme 2: internalizing honesty

All participants mentioned that honesty is a virtue for nurses in any ethically challenging situation. Many such situations would go unnoticed by others, even if nurses were dishonest about them. High workloads or situations in which others’ actions were difficult to observe directly often presented participants with the opportunity to make questionable choices for convenience rather than for the sake of honesty. Still, participants mentioned that they continually strove to internalize a sense of honesty to avoid unethical temptations.

*“The most important thing is honesty. I had to get ready for surgery really quickly, but then my collar or something touched my hand. I was afraid I’d get in trouble if I told my senior. However, it’s wrong to pretend that nothing happened. That’s why I always say I should not pretend that I do not know. I also tell other nurses not to do that” (Participant X, 12 years and three months of experience, operating department).*

### Theme 3: actively acting as an advocate

#### Sub-theme 1: expressing oneself regarding a treatment given or an erroneous situation

Participants actively participated in rounds with doctors and expressed their opinions on the treatment being given. Expert nurses accurately conveyed their opinions with confidence in conversations with doctors. For example, some participants asked the physician whether they had made a DNR decision too early. They also actively sought out physicians to solve problems (e.g., prescription errors). *“Even with dyspnea, I said to the doctor, ‘The current situation is not just an observation. The patient is showing Cheyne-Stokes breathing. You should come right away’”(Participant D, seven years and 10 months of experience, medical department).*

When nurses and doctors had difficulty communicating, participants would intercede to avoid any harm to the patient and various other ethical and legal problems (e.g., negligence of duty). Moreover, if fellow nurses or doctors did something wrong, participants mentioned it to the medical staff involved and asked them to rectify the mistakes. When participants became team leaders, preceptors, and charge nurses, they began to more actively check for unethical behavior, telling superiors and clinical professors, as well as other nurses, in advance about actions that might harm patient safety to prevent problems from occurring.

*“When educating family members to wear gowns ... Now I have a voice, and I have been working as a responsible nurse since last year. I am trying to do as much as I can” (Participant L, 11 years and six months of experience, surgical department).*

For situations in which problem-solving was not possible at the personal level, participants acted at the organizational level by reporting the problem to the head nurse (i.e., the unit manager), and then collecting and analyzing data on recurring problematic situations and reporting the results to the department of nursing administration.

#### Sub-theme 2: being a mediator

Participants helped in accurately conveying the opinions of the patient and family members to the physician during rounds, which helps maintain patients’ self-determination in therapy. One participant even asked another participant to suspend a DNR order until it had been sufficiently explained to patients and their family members.

The participants served as advisors to patients and family members to help them make the right choices. For example, a patient, who wanted to keep her illness a secret, raised the ethical issue of whether her mother should know of her medical condition. After listening to the patient, the participant recognized that the patient was suffering because of a broken relationship with her mother, and thus actively mediated the problem as a patient advocate. Another participant reported how a patient had been prescribed a medicine that was convenient for medical staff but financially burdensome to the patient, so she asked the doctor to correct the prescription by explaining the patient’s situation.

*“The only persons who can stand up for patients are nurses. We are their spokesmen. When I looked up the child’s past and current medical history, the child’s father was unemployed. I then found that the price of medicine, even though it was insured, was 47,000 won ($46). ... I asked the doctor to prescribe only the exact dose that was necessary”(Participant V, 13 years and five months of experience, intensive care unit).*

## Discussion

This study explored expert nurses’ experience of coping with ethically challenging situations to understand nurses’ ethical competence in nursing practice. The findings of this study suggest attributes of ethical competence that are mostly consistent with those of ethical competence as it has been previously defined in healthcare [4, 17, 18]. Eriksson et al. [17] argued that ethical competence in healthcare must consist of being (virtues), doing (rules and principles), and knowing (critical reflection). The findings of this study explained the characteristics of doing and knowing in greater detail than Eriksson et al. [17]. The expert nurses of this study expressed their “knowing” as they were self-monitoring for ethical insensitivity through self-reflection and ethical questioning with an awareness of human dignity. Gallagher [18] defined ethical competence as the possession of ethical knowledge, perception, reflection, and behavior. In this study, ethical knowledge was involved in the entire process of recognizing, reflecting upon, and acting on ethical issues rather than expressed independently from ethical perception, reflection, and behavior. For example, for a nurse to adhere to the standards of nursing, they must first know the nursing standards.

All participants of this study also expressed ethical perception as an awareness of human dignity in ethically challenging situations. Gastmans ([19], p.146) proposed that the essence of nursing care is “to provide care in response to the vulnerability of a human being in order to maintain, protect, and promote his or her dignity as much as possible.” Nurses have an ethical obligation to maintain and respect individuals’ dignity and integrity [20]. All participants realized the importance of preserving patients’ dignity, which in turn made them continually reflect on what the correct course of action was in ethically challenging situations.

Ethical reflection is an essential attribute of ethical competence [17, 18]. Using self-reflection and ethical questioning, all participants in this study habitually monitored their actions and thoughts to avoid ethical insensitivity while working in clinical settings. Ethical insensitivity or numbness is considered to be a loss of moral sensitivity [21] and is believed to be one of the main ethical issues that nurses face [22]. Nurses may become ethically insensitive when they are too busy with work in clinical settings [20, 22]. However, the participants in this study engaged in constant self-reflection to avoid ethical insensitivity. Self-awareness has been found to lead to greater competence in nursing [23]. Moreover, self-reflective behavior (e.g., asking oneself “What should I do?”) was also helpful in making nurses’ decision-making more ethical [24]. Nurses should engage in self-reflective behavior to be more ethically competent, and self-reflection could be encouraged through conversation [17] with colleagues or reflexive writing [24].

The expert nurses in this study mentioned the following types of ethical behaviors: adhering to principles and standards of nursing, internalizing honesty, giving one’s opinion on a treatment given or an erroneous situation, and being a mediator. Nurses have a responsibility to advocate for patients and their relatives, and are in an excellent position to be aware of the risk factors of ethical problems that may harm patients in clinical settings [25]. The participants could correct mistakes and resolve ethical conflicts by actively expressing their views on patients’ care and treatment by adhering to their own moral beliefs. Most importantly, nurses must be given opportunities to voice their opinions in ethically challenging situations to advocate for patients’ rights to health [26], and they require resources for dealing with their ethical quandaries [3]. Therefore, system-level support aimed at enhancing the moral resilience of nurses is necessary [27] for an ethical work environment [28].

## Limitations

Qualitative research is never intended to allow for generalization, but it is necessary to obtain a sufficient number of participants to describe a phenomenon comprehensively. In this respect, a limitation of this study is that expert nurses working in one general hospital in South Korea, rather than several hospitals, were included in the study. Because the study was only conducted in one hospital, despite the first and best hospital with a systematic career evaluation system for nurses in South Korea, the study results may not represent other hospitals in South Korea. Another limitation concerns the selection criteria for expert nurses—we relied on the hospital’s evaluation system, which is not nationally standardized; in the future, the criteria for defining an expert nurse need to be universally standardized. The authors did not consider the participant’s characteristics such as age or experience when forming the small interview groups, but mainly considered the participant’s available hours for interviews. Moreover, this study did not explore the role of the characteristic traits of expert nurses in coping with ethically challenging situations. Therefore, future research needs to study coping strategies by considering the moral character of nurses.

## Conclusions

The results of this study suggest that the best way for expert nurses to cope with ethically challenging situations is to care for patients faithfully according to the spirit of caring. After all, effectively coping with ethical issues requires nurses to realize the need to preserve the dignity of the patient, engage in self-reflection and ethical questioning, and maintain honesty as much as possible by adhering to the nursing principles and standards and speaking out as patient advocates. The results of this study could help nurses cope with the ethical difficulties they experience. Also, expert nurses’ experience of dealing with ethically challenging situations could help in guiding new nurses and nursing students. Furthermore, system-wide early counselling and interventions should be established for nurses who have experienced ethical difficulties to ensure ethical nursing practices.

## Data Availability

The datasets generated and/or analyzed during the current study are not publicly available due to the small sample size, but anonymized data are available from the corresponding author on reasonable request.

## Abbreviation

DNR: Do not resucitate order
